# Drug shortages in low- and middle-income countries: Colombia as a case study

**DOI:** 10.1186/s40545-022-00439-7

**Published:** 2022-06-13

**Authors:** Martha L. Sabogal De La Pava, Emily L. Tucker

**Affiliations:** 1 Department of Industrial Engineering, Clemson University, Clemson, SC USA; 2 School of Health Research, Clemson University, Clemson, SC USA

**Keywords:** Drug shortages, Colombia, Medicine availability, LMIC

## Abstract

**Background:**

Drug shortages are a global problem. Analyzing shortages worldwide is important to identify possible relationships between drug shortages across countries, determine strategies that reduce drug shortages, and reduce the inequality in access to medicines between countries. In contrast to well-documented shortages in high-income countries, there are few studies that consider low- and middle-income economies. We evaluate drug shortages in one middle-income country, Colombia.

**Methods:**

We collected data from INVIMA, the institution responsible for managing medicine shortage alerts in Colombia. We classified the data using the Anatomical Therapeutic Chemical (ATC) classification system and analyzed them using descriptive statistics. We considered a study period from 2015 to 2021 (vital medicines) and from 2010 to 2020 (non-vital medicines).

**Results:**

In total, 173 unique ATC codes were in shortage. These included antidotes, alimentary tract and metabolism products, anesthetics, cardiac stimulants and antithrombotic agents. The major causes were manufacturing problems and few suppliers. Drug shortages substantially increased from 2020 to May 2021 due to the COVID-19 pandemic. Among resolved shortages, the average duration was 1.6 years with a standard deviation of 1.9 years. The longest, naloxone tablets, were in shortage for almost 10 years.

**Conclusions:**

Drug shortages are a persistent problem in Colombia. Government institutions have made progress in implementing systems and procedures to report them. However, the approaches implemented need to be maintained and refined. This study lays the groundwork for the analysis of drug shortages in other LMICs. We highlight the necessity of addressing drug shortages in their global context and reducing the inequality in access to medicines between countries.

## Background

Drug shortages are a global problem, and many countries have struggled to maintain a consistent supply of medications. The United States has experienced hundreds of drug shortages in the past decade [1]. While high-, middle- and low-income countries all deal with shortages [2], documentation of shortages has focused on high-income countries (HICs) [3]. There are hundreds of papers about US shortages [4]. Yet there has been very limited work to understand the prevalence of shortages in low- and middle-income countries (LMICs).

When shortages occur, there may be serious impacts on patients and health systems. Health effects can include non-treatment, under-treatment, and errors occurring from the usage of alternative drugs [5]. Managing drug shortages and procuring alternatives can cost hundreds of millions of dollars annually for HICs [6].

Even in non-shortage conditions, health inequities persist between HICs and LMICs. Life expectancy is higher in HICs [7]. Within Colombia, life expectancy for children with acute lymphoblastic leukemia is 50–60% as compared to 90% in HICs [8]. When the antineoplastic medications used to treat it are in shortage, as they are currently, this only compounds of problem of lowered survival of children with cancer compared to HICs.

LMICs may be particularly susceptible to drug shortages. Without domestic manufacturing capacity, some countries have a strong reliance on the external supply of medicines [9, 10]. Within Latin America and the Caribbean, there is a persistent pharmaceutical trade deficit, i.e., current imports are approximately six times exports [11]. When these preexisting conditions combined with catastrophic or disaster situations such as disruptions from the COVID-19 pandemic, drug shortages can occur.

There has been support for improving medicine access globally, and one key dimension is availability of drug products [12]. However, while barriers to access have been studied at local levels [13–15], there are few country-level studies of drug availability and shortage in LMICs; studies that do exist focus on Asian and African countries [3]. In Colombia, as well as in other Latin American countries, an overview of country-level drug shortages is not available in English. Existing studies are primarily in Spanish and tend to either focus on specific drugs, e.g., shortage of cancer drugs [16] or study particular factors, e.g., prices and market concentration [17].

In this study, we provide a country-level overview of drug shortages in Colombia for 2010–2021. We jointly analyze the two sources of publicly available information for vital and non-vital unavailable medicines. We determine drugs affected, causes, durations, and discuss drug shortages in LMICs, generally. We aim to provide an overview to facilitate a better understanding of the global context of drug access and encourage further analyses of policies that could reduce shortages in LMICs.

### Colombia drug shortages reporting system

In Colombia, a drug shortage is defined as insufficient supply to satisfy the demand of any medicine approved and marketed in the country [18]. In 2018, the National Institute of Food and Drug Surveillance (INVIMA by its acronym in Spanish), was tasked as the institution responsible for managing the drug shortage alerts and implementing prevention and mitigation actions in Colombia [19]. Prior to 2018, it was a shared responsibility between the INVIMA and the Ministry of Health and Social Protection. Though Colombia lacks a sophisticated medicine shortage reporting system, users can report drug shortages to INVIMA through an electronic form. Using these warnings, the INVIMA confirms and manages the alerts and disseminates the information on their website.

One action to mitigate shortages was to declare some medicines as ‘vital unavailable medicines’ [19]. These are defined as (1) the essential and irreplaceable medicines to safeguard the life or alleviate a patient’s suffering and that, due to conditions of low profitability in their commercialization are (2) either not available in the country or are available in insufficient quantities [20]. Drugs in this category are subject to exceptions to import regulations, and the approval processes for manufacturing and selling the medications in the country are prioritized. Other drugs may also be short; we designate these ‘non-vital’.

Two types of drug shortage reports are publicly available from INVIMA. A single, general report includes information about vital unavailable and non-vital medicines, and a series of reports that provide updates on vital unavailable medicines specifically. The general shortage report was published on June 8, 2020 and includes information for 2010 (one reported case) and 2013 to 2020, i.e., drug name, warning date, revision date, state, causes, end date, and comments about the cases [21]. The states of shortage warnings are classified in: (1) Shortage, when it is confirmed that the availability of the medicine is insufficient with respect to the demand, also called in this study as active or open case; (2) monitoring, when they are observing, reviewing, and monitoring the availability of the drug. The latter is only included for the analysis of causes. (3) Closed, when the case is resolved. The second source is the collection of reports for vital unavailable medicines that present the list of shortages at the time of publication. Twenty reports are available from 2015 to 2021 and include the active ingredient name, pharmaceutical form, concentration, pharmacological norm, minutes number, and national code [22].

## Methods

We first integrated the two sources (general report and collection of vital unavailable medicine reports). In each, we appended the appropriate ATC code to each record using the drug name. It was not a one-to-one match; some records had slightly different drug names and corresponded to the same ATC code. Eight drugs in the general report could not be matched with an ATC code, and these were excluded from ATC code-specific analyses. To calculate the shortage duration, for the first source (general report), we used the warning and end dates. For the collection of reports for vital unavailable medicines, we approximated the duration as follows. Each report in the collection provided the list of drugs currently in shortage. We recorded the date of the report the medicine first appeared in the list and the date of the report when the drug was no longer listed. After implementing the above, the integrated database includes the following information: ATC code, drug name, state (closed or active), causes, initial date, end date, exact or approximate duration, report date, and data source. Descriptive statistics were used to analyze the integrated database. In addition, the vital unavailable medicines reports were studied independently with the same tool. Results are presented using the combined database, except where directly noted.

## Results

In total, there were 229 reported shortages from 2010 to 2021 (219 unique drugs). Ten drugs were short two or more times during the study period. The most frequent chemical subgroups that have been in shortage are shown in Table 1. Among all shortages (left-hand side), the most common categories represent 25% of all shortages. The right-hand side considers the 173 ATC codes with active shortages and represents approximately 34% of the open cases. The most frequent categories of active cases overlap with frequent categories of all cases, though the ranking order may vary. In both, antidotes are the most common category of shortages ($n=10$ among all, and $n=9$ among active). Other drugs included in both listings are local and general anesthetics, cardiac stimulants, antithrombotic agents, medical gases, and muscle relaxants and peripherally acting agents. Three additional elements occur commonly in the active cases; these are the antiseptics and disinfectants, the immune sera, and the antineoplastic agents.

**Table 1:** Top five of the most frequent chemical subgroup in shortage

All cases, closed and active (*N* = 229)				Active cases (*N* = 173)			
No.	Chemical subgroup	*n*	%	No.	Chemical subgroup	*n*	%
1	Antidotes	10	4.4%	1	Antidotes	9	5.2%
2	Amides	8	3.5%	2	Various alimentary tract and metabolism prods	6	3.5%
3	Enzymes	6	2.6%	3	Enzymes	5	2.9%
	Various alimentary tract and metabolism prods	6	2.6%		Heparin group	5	2.9%
					Amides	5	2.9%
4	Amino acids and derivatives	5	2.2%	4	Adrenergic and dopaminergic agents	4	2.3%
	Heparin group	5	2.2%		Other quaternary ammonium compounds	4	2.3%
	Adrenergic and dopaminergic agents	5	2.2%		Other general anesthetics	4	2.3%
	Other general anesthetics	5	2.2%		Medical gases	4	2.3%
5	Other quaternary ammonium compounds	4	1.7%	5	Amino acids and derivatives	3	1.7%
	Medical gases	4	1.7%		Other antiseptics and disinfectants	3	1.7%
					Immune sera	3	1.7%
					Other antineoplastic agents	3	1.7%

**Fig. 1:**
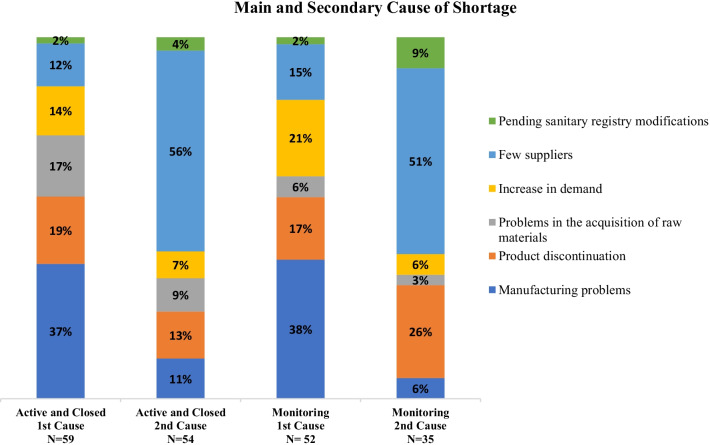
Main and secondary cause of shortage: active and closed states; monitoring state (based on the shortages with an identified cause)

In most of the cases the reason for shortage is unknown. The main cause was provided for 59 out of 229 shortages. In 37% of the cases with identified causes, the main cause of the shortage is manufacturing problems, which corresponds to issues with the current Good Manufacturing Practice certification. In 19%, the main cause is product discontinuation, which is the decision of the sanitary registration holders not to continue trading or selling the medicine. In addition, a secondary cause was reported for 54 cases, where 56% was due to low number of suppliers. This suggests that while the number of suppliers may not be the primary cause of shortages, not having backup suppliers can exacerbate other supply chain issues. The distribution of causes can be seen in Fig. 1 (first and second bars).

The general shortage report also presented drugs that are being monitored for potential shortage. For the 52 monitoring cases, the most frequent cause was the same as the active and closed states (manufacturing problems). However, the other positions changed; the second most frequent was increases in demand. For monitoring cases with a second known cause, few suppliers and product discontinuation are the second leading causes, consistent with active and closed states in the integrated database.

### Vital unavailable medicines

Next, we will consider vital unavailable medicines specifically. Currently, there are 399 drugs classified as vital unavailable medicines (report published on May 07, 2021). This corresponds to 162 ATC codes. Across the entire study period (2015 to May 2021), a total of 187 different ATC codes have been classified as vital unavailable. Six drugs have cycles, i.e., they have entered, left, and reentered the list. These medicines are betaine, fibrinogen, isoprenaline, methotrexate, suxamethonium, and pyrimethamine. The current state of the last drug is resolved; the others are active shortages.

**Fig. 2:**
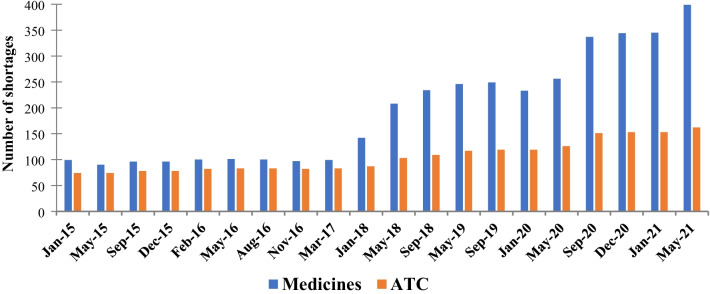
Number of vital unavailable medicines over time

The number of vital unavailable drugs over time can be seen in Fig. 2. The orange bars represent the number of ATC codes, and the blue bars the number of medicines. Medicines are defined consistently with the vital unavailable drug reports; it is a narrower category than ATC codes that designate active ingredient, concentration, and pharmaceutical form. A large increase in the number of vital unavailable medicines occurred in 2018; 92 new medications entered the list, equivalent to 64.8% increase. Two other substantial increases occurred from June to September 2020 with 81 new medications (+ 31.6%) and from February to May 2021 with 54 new medicines (+ 15.7%).

The COVID-19 pandemic has affected the list of medications that are considered vital unavailable. Drugs needed to prevent, diagnose, and treat COVID-19 patients or whose global supply chains have been affected by cancellation or restrictions from the pandemic were added to this category [23]. Of the new medicines classified as vital unavailable in the last five reports (May 2020–May 2021), 43%, 28%, 71%, 0% and 24% correspond to the COVID-19 pandemic, respectively. Most of these medicines are medical gases; antiseptics and disinfectants; antithrombotic agents; cardiac stimulants; general anesthetics such as nitrous oxide, ketamine hydrochloride, etomidate and propofol; and muscle relaxants used to facilitate endotracheal intubation and provide skeletal muscle relaxation during surgery or mechanical ventilation.

### Duration of drug shortages

**Fig. 3:**
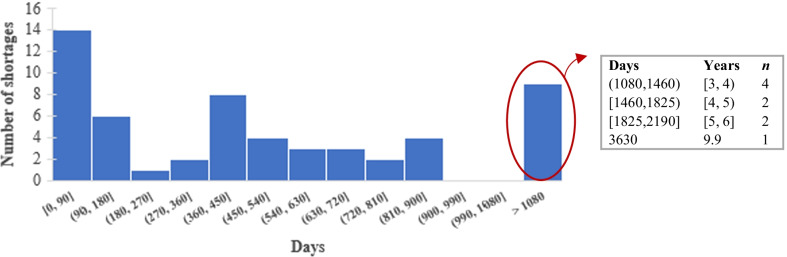
Histogram of shortage duration—closed cases (ATC)

To analyze the duration of drug shortages, we separated the closed and active cases. Figure 3 presents the duration of the 56 resolved shortages. Approximately 36% of the cases were in shortage between 1 day and 6 months. Nine cases (16%) were in shortage for more than 3 years, of which eight are vital unavailable medicines. Naloxone in tablets was short for almost 10 years due to an increase in demand and few suppliers. The approximate average drug shortage duration for closed cases is 1.6 years with a standard deviation of 1.9 years.

**Fig. 4:**
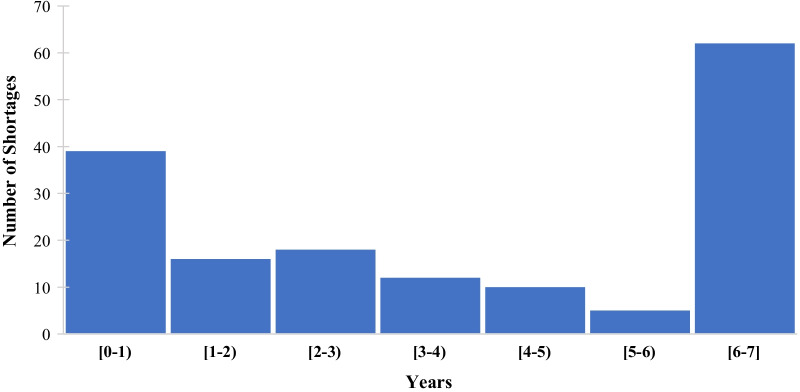
Approximate shortage duration: vital unavailable medicines—active cases (ATC)

For active drug shortages, we separated vital and general drugs. The vital unavailable shortage durations are approximate (Fig. 4), and the general shortage durations are exact. The average vital unavailable duration is 3.9 years with a standard deviation of 2.4 years. Among ATC codes from the first report (Jan. 2015), 62 remained listed on the last report (May 2021); approximately 38% of the vital medicines’ active cases have been on the list for 6.4 years.

Among general drugs, there are 11 unresolved shortages (report date June 2020) which have been in short on average for 1.1 years (standard deviation 1.6 years). Nine cases have been in shortage for less than 1 year. Calcium folinate 15 mg in tablet has been short for 5.8 years because no company is interested in selling the drug in Colombia.

## Discussion

We provide an overview of the reported drug shortages in Colombia from 2010 to 2021. By quantifying these shortages, healthcare providers, regulators, and companies can better identify strategies to mitigate them.

Our results suggest that drug shortages in Colombia are common and persistent. Traditional global health concerns focus on reducing the prevalence of communicable diseases (CDs) worldwide. Many strategies have been implemented to provide access to medicines in all countries to treat CDs compared to noncommunicable diseases (NCDs) [24], which are the leading cause of death in the world. Around 82% of the early deaths caused by NCDs occurred in LMICs [25]. Chronic NCDs are the main cause of morbidity and mortality in Colombia; hypertension, diabetes, asthma, coronary heart disease, stroke, and cancer are the most prevalent chronic conditions [26]. Many drugs used to treat NCDs have been in shortage. These include vinblastine, vincristine, cyclophosphamide, idarubicin, and tamoxifen which are used to treat cancer. Epinephrine, used to treat anaphylaxis, cardiac arrest, and severe asthma attacks, and propafenone used to treat arrhythmia have also been short. Knowing and understanding drug shortages in each country would help evaluate to what extent drug shortages contribute to the mortality or worsening of the health condition of patients suffering NCDs.

Vital unavailable medicines emerge as a strategy to provide access to medications that are difficult to obtain in the country, either due to low demand, low profitability, or stock-out because of crisis times, as was seen with the COVID-19 pandemic. Declaring a drug as a vital unavailable gives a path to respond to drug shortages. However, it does not solve the structural problems of the Colombian market related to these medicines. That is, the drug will continue to be in short supply in the country.

The Colombian government has published a database with the list of approved import requests for vital unavailable medicines. The INVIMA is responsible for authorizing the importation of these medicines to requesting importing companies, who must specify whether the importation of the drug is for a particular patient, a group of patients, or a clinical emergency [27]. Considering the last 5 years (2018 to May 2022), the three most requested approved active ingredients were ataluren to treat muscular dystrophy, burosumab to treat disorders of phosphate metabolism and rickets, and lutetium-177 to treat malignant tumors. These represent 14.5% of the 3962 approved import requests received in that period.

In addition, we found that anesthetic drugs are among the top five most frequent shortages in Colombia. Current anesthetic shortages are primarily caused by the COVID-19 pandemic. These shortages have safety and cost implications for patients and institutions because they lead to the cancellation of procedures [28].

There are challenges to effective synthesis of drug shortage data in Colombia. Publicly available data on drug shortages are not updated in real-time, and data collection was not standardized prior to 2018. This combination suggests that the drug shortage situation in Colombia may be worse than the results presented. To observe the full context, the structural changes that were made in 2018 to improve the reporting of drug shortages should be maintained and expanded. These would also support comparative analyses in the future.

Comparing drug shortages between countries is difficult given the low standardization in the reporting procedures and the lack of national institutions reporting shortages in some countries [29]. To understand shortages from a global perspective, further information is needed on drug shortages in other LMICs. To facilitate this, each country could implement a centralized shortage reporting system [30]. It should be standardized and harmonized according to the best practices [2, 31]. We note that collaborations with researchers who are familiar with each system are key.

The limited study about drug shortages in Colombia contrasts with the understanding of shortages in high-income countries. There are several studies about drug shortages in the US and the European Union [3, 4]. The European Economic Area promotes the exchange of information and best practices on the availability of medicines in each country through the network Heads of Medicines Agencies.

A cross-country understanding of drug shortages is critical because the world economy works under globalized supply chains. Within the pharmaceutical market, suppliers, manufacturing plants, distribution centers, and patients are around the world. Events that lead to drug shortages in one country could impact the supply of medicines in other countries. Quantifying drug shortages in many countries would help identify how shortages in different geographical regions are related and identify availability gaps across countries. These analyses are necessary to create strategies to ensure worldwide drug access as part of the universal health coverage goal.

### Limitations

The analyses were performed using INVIMA data which are not updated frequently. The shortage durations of the vital unavailable medicines are approximations because they are based on the dates of publication of the reports. Also, the databases do not provide the ATC code, a necessary element to integrate the data with other countries’ shortages. The matching process to link drug names with the ATC codes was done by hand and using the Colombian pharmacological standards published in June 2019 [32]. Despite these limitations, this overview serves as a starting point to a deeper study of the drug shortage situation in Colombia and its integration with global data.

## Conclusion

This study is the first English overview on drug shortages in Colombia and considers the two publicly available data sources on general and vital unavailable medicines. The analysis contributes to information about drug shortages in a South American middle-income country that can be integrated into global analyses. It also lays the groundwork for the study of drug shortages in other LMICs. A wide range of medicines have been in shortage in Colombia. Some of these drugs are in the World Health Organization Model List of Essential Medicines. Shortages have primarily been caused by manufacturing problems and few suppliers. From 2020 to May 2021, 166 medications became vital unavailable medicines, of which 51 are due to the COVID-19 pandemic. Future work should be addressed to quantify drug shortages in other LMICs, analyze and compare drug shortages between LMICs and HICs. These analyses would allow researchers to approach medicine availability from a world equity perspective.

## Data Availability

The datasets used in the current study are available upon request.

## Abbreviations

ATC: Anatomical therapeutic chemical
CDs: Communicable diseases
HICs: High-income countries
LMICs: Low- and middle-income countries
NCDs: Noncommunicable diseases
